# Microwaves, a potential treatment for bacteria: A review

**DOI:** 10.3389/fmicb.2022.888266

**Published:** 2022-07-25

**Authors:** Zhen Zhang, Jiahao Wang, Yihe Hu, Long Wang

**Affiliations:** ^1^Department of Orthopedics, Xiangya Hospital, Central South University, Changsha, China; ^2^Hunan Engineering Research Center of Biomedical Metal and Ceramic Implants, Xiangya Hospital, Central South University, Changsha, China; ^3^Department of Orthopedics, First Affiliated Hospital, School of Medicine, Zhejiang University, Hangzhou, China; ^4^National Clinical Research Center for Geriatric Disorders, Xiangya Hospital, Central South University, Changsha, China; ^5^Hunan Key Laboratory of Aging Biology, Xiangya Hospital, Central South University, Changsha, China

**Keywords:** microwave, bacteria, application, biosensor, mechanism

## Abstract

Bacteria have brought great harm to the public, especially after the emergence of multidrug-resistant bacteria. This has rendered traditional antibiotic therapy ineffective. In recent years, hyperthermia has offered new treatments to remove bacteria. Microwaves (MW) are a component of the electromagnetic spectrum and can rapidly heat materials. Taking advantage of this characteristic of MW, related studies have shown that both thermal and non-thermal effects of MW can inactivate various bacteria. Even though the understanding of MW in the field of bacteria is not sufficient for widespread use at present, MW has performed well in dealing with microorganisms and controlling infection. This review will focus on the application of MW in bacteria and discuss the advantages, prospects and challenges of using MW in the bacterial field.

## Introduction

Bacteria have threatened human health for thousands of years (Fournier et al., 2013), and antibiotic treatment has been used for antibacterial activity. However, with the overuse and inappropriate use of antibiotics, bacterial multidrug resistance has emerged, making it difficult to control and eliminate bacterial infections (Ghosh et al., 2019; Monserrat-Martinez et al., 2019). Bacterial infections are a serious challenge, with 700,000 patients currently dying each year from drug-resistant infections, and the total number of deaths will rise to 10 million per year from 2050 if urgent attention is not given (Tagliabue and Rappuoli, 2018). Sadly, this tragedy is difficult to reverse because biofilms protect bacteria and promote their resistance, and the development of antibacterial methods lags behind bacterial resistance. Biofilms are a major problem for antibacterial methods (Hathroubi et al., 2017; Yin et al., 2019). Biofilms are an advanced form of microbial community protection in which bacterial cells can significantly evade host immune responses, mediate desiccation tolerance and resist antimicrobial therapy, causing animal and plant diseases and threatening medical infections (Holmes et al., 2016; Hathroubi et al., 2017; Wood, 2017; Yin et al., 2019). Currently, at least two-thirds of clinical infections are associated with biofilms, and these patients cannot be cured by antibiotic therapy alone (Wolfmeier et al., 2018; Dostert et al., 2019). Furthermore although many antibacterial methods have been developed (Shi et al., 2016; Sun et al., 2018; Ghosh et al., 2019; Chen et al., 2021; Wang and Sun, 2021) in the past few years, including near-infrared (NIR) light irradiation (Han et al., 2021), ultrasonic irradiation (Rezek Jambrak et al., 2018; Shu et al., 2021), pulsed electric fields (PEF) (Gabrić et al., 2018; Garner, 2019), nanoparticle-based topical antimicrobial drug delivery (Gao et al., 2018; Van Giau et al., 2019), nanomaterials (Makabenta et al., 2021), antimicrobial/biofilm peptides (Dostert et al., 2019), and bacteriophages, bacterial resistance extends beyond antibiotic use (Ghosh et al., 2019), encompassing changes in temperature (Ojha et al., 2016), nutrient limitation (Maisonneuve et al., 2018), pH (Helaine et al., 2014), and other factors (Fisher et al., 2017). Once resistance is obtained, new resistant strains will gradually replace susceptible strains (Holmes et al., 2016). Therefore, new methods to eliminate bacterial infections are urgently needed.

In recent years, microwaves (MWs) have performed well in the treatment of tumors, and have become a relatively novel and popular tumor treatment method (Ruiter et al., 2019; Cazzato et al., 2021). However, their ability to eliminate bacterial infections has not received enough attention. MW as a component of the electromagnetic spectrum, are non-ionizing radiation with frequencies ranging from 300 MHz to 300 GHz, and wavelengths between 1 m and 1 mm (Mawioo et al., 2016; Wang et al., 2019a; Shaw et al., 2021). The effects of MW include thermal and non-thermal (Rougier et al., 2014; Shaw et al., 2021), and it is generally believed that the temperature increase caused by MW exposure plays a key role in microorganism inactivation (Rougier et al., 2014; Shaw et al., 2021). The MW-based thermal effect is different from conventional heating (Shaw et al., 2021), involving the dielectric properties of polar substance molecules, which have a shorter heating time than conventional heating. More importantly, MW-based non-thermal effects can destroy microorganisms at temperatures below the thermal destruction point, which can disrupt cell membranes and increase the amount of DNA and proteins released from cells (Woo et al., 2000; Shaw et al., 2021). There are several advantages of MW, including traveling at the speed of light, delivering energy directly to objects, being easily to controlled, enabling deep penetration capabilities, rapidly heating, and causing negligible damage to healthy cells and tissues (Vogl et al., 2011; Zhang et al., 2017; Chen et al., 2020; Papini et al., 2020; Ricci et al., 2020; Weiss et al., 2020). Although the inactivation ability of MW to microorganisms has been summarized in some reviews (Cui et al., 2022; Skowron et al., 2022), the content is inclined to the treatment of food and liquid by MW. This review is more inclined to the role of MW in the field of biomedicine.

Therefore, to better understand the infection controlling ability of microwaves and to broaden the horizon on microwave clinical utility, this article will review the current status, existing problems and prospects of MW elimination of bacterial infections.

## Application of microwaves in controlling bacteria

### Inactivated bacteria

Most pathogenic bacteria can multiply between 33 and 41°C (Mackowiak, 1981). When the temperature increases, the proliferation and mobility of bacteria are inhibited (Ibelli et al., 2018). Studies (Tsuchido et al., 1985; Menezes and Teixeira, 1992; Ibelli et al., 2018) have shown that the outer membrane of gram-negative *Escherichia coli* undergoes reversible disruption above 46°C, and furthermore, exposure of *E. coli* to 45°C for 10 min reduces protein synthesis. Due to the dipolar nature of water, when water is exposed to MW, the dipolar water molecules rearrange in the direction of the electric field. The high frequency of MW induces billions of oscillations per second of intracellular ions and polar molecules, and its intense friction causes very rapid heating. Therefore, microbial inactivation by MW is mainly concentrated in liquids, solids, and food with a moisture content higher than 50%. In recent studies, MW has been used in other situations, such as airborne microbial inactivation (Table 1).

**TABLE 1:** A summary of the destruction of bacteria by MW *in vitro*.

Experimental setup						
MW frequency (GHz)	Energy/power	Exposure temperature (°C)	Exposure time	Biological target/object	Effects	References
–	700 W	100	3 min	Retorted vegetables	MW reduced the bacteria level by 10^3^CFU/g.	Cho and Chung, 2020
0.915	6 kW	90	5 min	Peanut butter	*L. monocytogenes*, *E. coli* O157:H7 and *S. Typhimurium* in peanut butter was reduced.	Song and Kang, 2016
2.45	600 W	100	5 min	Sludge	MW pretreatment could remove 13.5–35.5% of ARBs in the pH range down from 10 to 2.5.	Tong et al., 2016
2.45	465 W	71	1 min	Sludge	After the exposure to MW irradiation, in a 20 g sludge sample, the concentration of *E. Coli* decreased to below than analytical detection levels.	Mawioo et al., 2016
–	260 W/m^3^	100	20 s	*E. coli*	MW irradiation induced airborne *E. coli* lysis of 4.1 log reduction in 20 s.	Wang et al., 2019b
2.45	800 W	25–100	1 min	*C. difficile* spore	After MW treatment, *C. difficile* spore complete inactivation in aqueous suspension at 10^7^ CFU/ml.	Ojha et al., 2016
2.45	700 W	–	1.5 min	*B. subtilis* spores and *Pseudomonas fluorescens*	Under MW irradiation, only 35% of *B. subtilis* spores survived and 5.8% of *Pseudomonas fluorescens* survived.	Wu and Yao, 2010
2.45	750 W	–	1.5 min	*B. subtilis* spores	*B. subtilis* spores achieved 3 log disinfection.	Zhang et al., 2010
2.45	500 W	–	1.5 min	*E. coli*	*E. coli* under the detection limit.	Zhang et al., 2010
4.592	650 W	–	3 min	Polymethyl methacrylate disks	MW combination for 3 min reduced *C. albicans* biofilm formation.	Martinez-Serna et al., 2021
2.45	150 W	70–110	5 min	*Bacillus cereus* biofilms	MW irradiation achieved complete inactivation of *Bacillus cereus* biofilms.	Park et al., 2017

#### Solid medium

Beginning in the 21st century, increasing attention was paid to food safety and nutrition (Pollock et al., 2017). Pathogens in food cause many diseases, especially gastrointestinal diseases (Boyle et al., 2007; Robertson et al., 2016). As a common method to control foodborne pathogens, conventional thermal treatment destroys physicochemical and sensory properties (Mandal et al., 2020), which has led to new processing methods that can ensure safety and preserve the nutritional and sensory quality of food products. One study (Cho and Chung, 2020) showed that MW can reduced the bacterial level by 10^3^CFU/g in retorted vegetables (700 W, 3 min), and uses less energy than steam (70–80%) at 100°C for 20 min. MW irradiation can simultaneously maintain good quality and control the levels of microorganisms in retorted vegetables. Song and Kang (2016) evaluated the efficacy of MW to inactivate pathogens in peanut butter. When treated with 6 kW MW for 5 min, the *L. monocytogenes*, *E. coli* O157:H7 and *S. typhimurium* in peanut butter were reduced (by more than 3 logs, 4 logs and 5 logs respectively). Moreover, the concentration of *S. typhimurium* was under the detection limit (1.0 log CFU/g).

In recent years, wastewater has been recognized as a major source of antibiotic resistance (Zhang et al., 2009; LaPara et al., 2011). Numerous studies have shown that sludge and biosolids from wastewater treatment plants contain many antibiotic-resistant bacteria (ARB; Munir et al., 2011; Naquin et al., 2015). A study by Tong et al. (2016) showed that pretreatment with MW could reduce ARB during sludge anaerobic digestion. The MW reactor was operated at 600 W with a stirring rate of 50 rpm, the reaction time was set to 5 min, and the heating rate was 16°C/min from 20 to 100°C. A total of 500 mL of egg-shaped digester inlet sludge samples was pretreated under different pH conditions. The results showed that MW pretreatment could remove 13.5–35.5% of ARBs (0.55–5.04 log) in the pH range from 10 to 2.5. Mawioo et al. (2016) showed that exposure of a 20 g sludge sample to MW irradiation reduced the concentration of *E. Coli* to below analytical detection levels (i.e., b1000 CFU/g TS), when samples were treated with 456 W for more than 1 min (i.e., MW energy = 8 Wh, temperature = 71°C). Moreover, this pathogen reduction was the time- and radiation power-dependent, which means that we can change the radiation power or exposure time to obtain the desired bacterial reduction. Generally, MW reduces bacteria in sludge with high efficiency and low energy consumption.

The COVID-19 pandemic has caused a severe and international shortage of filtering facepiece respirators. He et al. (2020), showed that MW irradiation achieved 100% bacterial inactivation (>4 log reduction) of *E. coli* and *B. subtilis* in 30 min, at 400 W output power (to avoid damage to the filtering facepiece respirators from prolonged exposure to MW, the single treatment time in the experiment was no more than 5 min), which is less time-consuming than 90 min of steam. Moreover, this filtering facepiece respirator regeneration did not affect the filtration performance. The quantified respirator fit and function preservation after MW irradiation were also reported in another study (Zulauf et al., 2020).

#### Air and water

**FIGURE 1:**
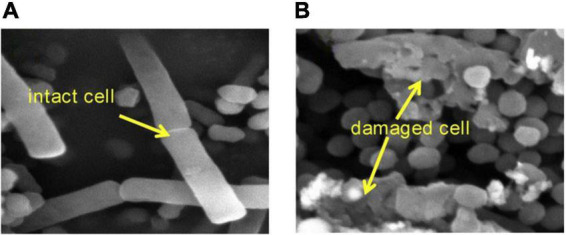
SEM images of *E. coli* **(A)** before MW irradiation and **(B)** after MW irradiation (Wang et al., 2019b).

Bioaerosols are airborne microbial cells with debris and particulate matter of any biological origin (Liang et al., 2012). These small particles can cause infectious diseases, acute toxic reactions and allergies (Gergen, 2001). Because of the pandemics of severe acute respiratory syndrome and influenza H1N1 viral infections, bioaerosols have attracted worldwide attention. Recently, one study (Wang et al., 2019b) showed that MW irradiation (260 W/m^3^) induced airborne *E. coli* lysis with a 4.1 log reduction in 20 s (Figure 1), releasing endotoxins from *E. coli* by heating. In addition, MW irradiation degraded endotoxins, achieving a 35% removal efficiency when the temperature increased to 200°C. In a further study (Wang et al., 2019a), MW irradiation showed nearly 20 times the inactivation rate of airborne *E. coli* than of waterborne *E. coli*, which was because water absorbed most of the MW energy (92.3%) to increase the temperature rather than kill bacteria. The more absorbed energy could be used to inactive airborne bacteria. Finally, *E. coli* requires 2.3 J and 116.9 J energy, respectively, for each log of inactivation of airborne and waterborne disease.

**FIGURE 2:**
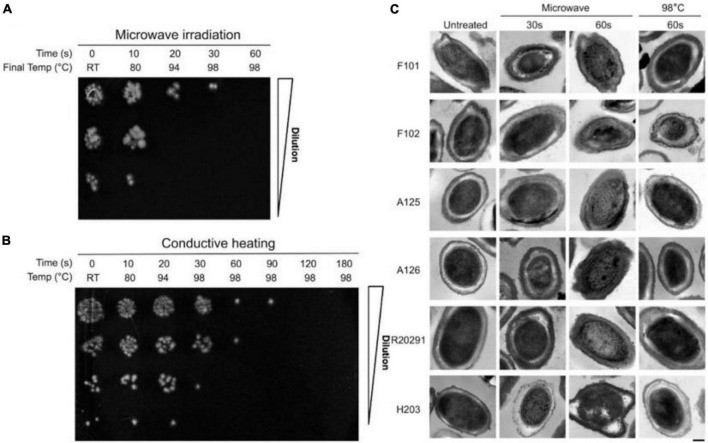
**(A)** Survival of *C. difficile* spores following microwave irradiation and conductive heating. **(B)** Survival of *C. difficile* spores following conductive heating. **(C)** Transmission electron micrographs of *C. difficile* spores following microwave irradiation and conductive heating (Ojha et al., 2016).

Spores act as vectors for bacteria, which can cause severe disease (Peng et al., 2018). The study showed (Ojha et al., 2016) that, after MW treatment at 800 W for 60 s, *C. difficile* spores were completely inactivated in an aqueous suspension at 10^7^ CFU/ml. However, this was not observed in conductively heated spores (Figure 2). Wu and Yao (2010) investigated the survival of bioaerosols after MW irradiation (2450 MHz, 700 W). Under 1.5 min MW irradiation, only 35% of *B. subtilis* spores survived, and 5.8% of *Pseudomonas fluorescens* survived. Another study (Zhang et al., 2010) compared the MW irradiation effects of *E. coli* and *B. subtilis* spore bioaerosols. The results showed that *B. subtilis* spores were more difficult to destroy and required irradiation at 750 W for 90 s to achieve three logs of disinfection. However, the viability of *E. coli* was below the detection limit at 500 W for 90 s.

One study (Kuo et al., 2016) showed that magnetic nanoparticles (MNPs) can selectively trap bacteria when exposed to MW heating for 60 s, which could be due to their high specific surface area and magnetic properties. In this way, *S. aureus* can be identified by matrix-assisted laser desorption/ionization mass spectrometry. However, this method can trap only a few tens of bacteria in a small sample (20 μL).

It is clear from the above studies that MW irradiation is a simple and time-saving treatment that inactivates airborne and water-suspended microorganisms, thereby reducing the risk of bacterial infection.

### Reducing antibiotic resistance

Antibiotic resistant bacteria have become a global health crisis, and China, as one of the world’s largest producers and consumers of antibiotics, is witnessing this crisis (Qiao et al., 2018). The overuse of antimicrobials and the enrichment of antibiotic resistance genes (ARGs) have caused the emergence of ARB as well as environmental protection. Antibiotics and ARGs are widely distributed in surface water, wastewater treatment plant effluent, soil and animal manure (Pruden et al., 2006; Qiao et al., 2018), which reduces the bacterial therapeutic potential in humans and animals (Wright, 2010). Therefore, it is urgent to develop a novel method for degrading ARGs and antibiotics in the environment.

One study (Kor-Bicakci et al., 2020) conducted research on triclosan (TCS), a persistent, poisonous, bioaccumulative antimicrobial found at high concentrations in wastewater (McAvoy et al., 2002). The results (Kor-Bicakci et al., 2020) showed that, compared with anaerobic digestion, MW pretreatment could easily degrade TCS (from lower than 25–46%), and a higher digestion temperature led to a higher degradation rate. Spiramycin, another high concentration antibiotic in wastewater (Zhu et al., 2014; Klein et al., 2018), can also be mitigated by MW. The related results from one study (Chen et al., 2019) showed that spiramycin (100 mg/L) was rapidly and completely removed after 8 min of reaction with silicotungstic acid under 200 W MW irradiation. MW irradiation alone removed 30.1%, and silicotungstic acid alone removed 15.9%. The degradation rates were positively correlated with MW power and interaction time. In another study (Tong et al., 2016), the relative concentration of ARGs was lower than that of sludge anaerobic digestion after combined MW (600 W) pretreatment.

### Destroying biofilms

In the food and medical industries, biofilms can cause serious problems (van Wolferen et al., 2018). Biofilms in processing equipment pose a threat to product safety and can lead to consumer health concerns (Alav et al., 2018). In clinical medicine, approximately 80% of chronic infections are associated with biofilms (Caputo et al., 2018), such as those in surgical implants. Elevated temperature resulted in a lower elastic modulus and stiffness of staphylococcal biofilms, which may be beneficial for biofilm removal (Ibelli et al., 2018). Many studies (Li et al., 2019; Elbourne et al., 2020; Wang Q et al., 2021) have shown that a magnetic field can effectively destroy biofilms, furthermore, magnetic hyperthermia can further affect biofilm damage. This effect can be found in both gram-positive and gram-negative bacterial biofilms (Elbourne et al., 2020), methicillin-resistant *Staphylococcus aureus* (MRSA) biofilms (Li et al., 2019), and *Staphylococcus aureus* and *Pseudomonas aeruginosa* biofilms (Wang Q et al., 2021).

**FIGURE 3:**
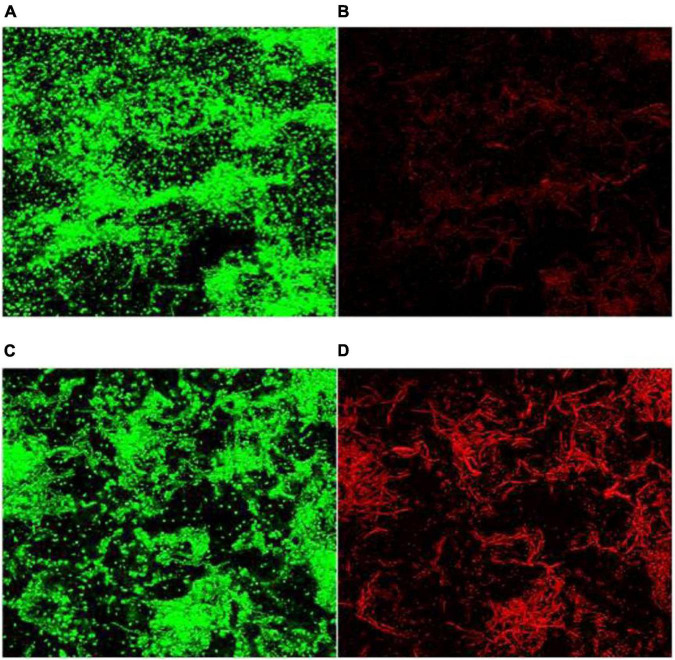
CLSM images (×40) of *B. cereus* spores in biofilms. **(A,B)** Before MW and **(C,D)** after microwave irradiation. Green fluorescence: living cells and dead cells; red fluorescence: dead cells (Park et al., 2017).

As a component of the electromagnetic spectrum, MW can generate electromagnetic fields, and the strength of the electromagnetic field is positively correlated with the frequency of the MW (Nguyen et al., 2015). One study (Martinez-Serna et al., 2021) tested whether hydrogen peroxide (H_2_O_2_) immersion and MW exposure could exhibit the antibacterial effect of *C. albicans* biofilms on the surface of polymethyl methacrylate discs, showing that H_2_O_2_ alone could not eliminate the formation of *C. albicans* biofilms; however, the 650 W MW combination for 3 min reduced *C. albicans* biofilm formation. With high resistance to physical and chemical treatments (Andersson et al., 1995). Park et al. (2017) showed that the inactivation of *Bacillus cereus* biofilms by conventional heating requires a temperature of 108°C for 15 min. MW irradiation (150 W) achieved complete inactivation within 5 min, which was observed by confocal laser scanning microscopy (CLSM) (Figure 3).

### Monitoring and identifying bacteria

In many clinical diseases (such as sepsis and prostheses joint infection), rapid treatment is associated with reduced mortality and high-quality functional recovery (Karam et al., 2016). However, bacterial diagnosis and antibiotic susceptibility testing (AST) are often delayed. Therefore, more rapid identification methods need to be developed to provide faster pathogen identification and AST, which can help optimize antibiotic prescribing, reduce resistance and prevent the spread of multidrug-resistance pathogens.

**FIGURE 4:**
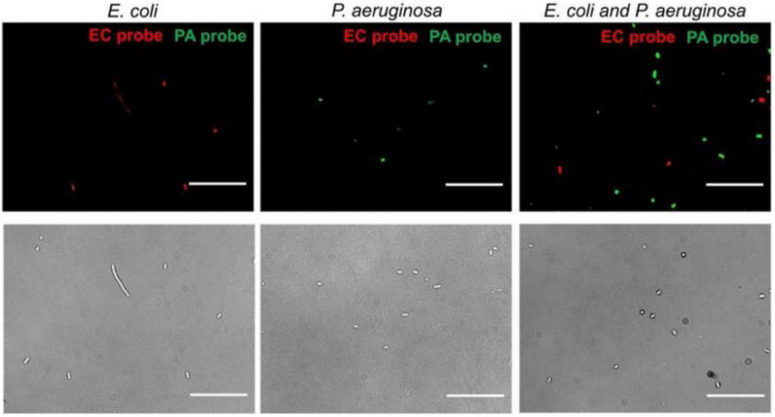
Multiplex detection of *E. coli* and *P. aeruginosa* by NAME (Gao et al., 2019).

Microwaves can be used to identify pathogens. Gao et al. (2019) designed a nanotube-assisted microwave electroporation (NAME) method. In this method, carbon nanotubes are used as sensors that absorb microwave energy, which induces electroporation in the cell wall. Through this electroporation, intracellular probes of double-stranded nucleic acids targeting specific bacterial 16S rRNA and fluorophores can be delivered in bacteria by MW irradiation (2.45 GHz, 30S), when the probes are fed into the bacteria, fluorescence microscopy can observe the specific fluorescence of different pathogens (Figure 4). NAME can identify pathogens, and all of the processes take only 30 min. On the basis of the electroporation properties of WM, it has also been used to specifically identify *Chlamydia trachomatis* (Zhang et al., 2011; Melendez et al., 2013) and non-typhoidal *Salmonella* (Tennant et al., 2011).

To achieve rapid AST, it is critical to have an instrument that can sensitively monitor bacterial proliferation. On this basis, AST can be achieved in a short time after coculture of antibiotics and bacteria. MW-based biosensors are a great tool, as the surrounding conductivity or loss tangent is constantly changing during bacterial growth, which results in a change in the resonance amplitude of the MW. One study (Jain et al., 2021a) showed a label-free and non-contact MW-based biosensor. This biosensor is a one-port microstrip ring resonator, and the operating frequency is 1.76 GHz, which can detect and monitor the growth of *E. Coli*. The perfect fitting between bacterial growth measurement by MW and the Gomperta growth model indicates that this MW-based biosensor has the potential for bacterial detection and monitoring. MW resonator sensors present the ability to monitor bacterial growth in liquid media, as well as in solid agar media. Another newly developed MW-based sensor (Mohammadi et al., 2020) showed good performance in monitoring the growth of *E. coli* on Luria-Bertani agar. The same result was found in another paper (Jain et al., 2021b), which illustrates that the MW split- ring resonator (1.76 GHz) monitors *E. coli* cultured on solid agar medium providing rapid, non-contact, invasive free sensing and monitoring. Jain et al. (2021b) conducted a further study on AST, and the results confirmed that AST can be monitored by adding the antibiotic to the solid agar medium where bacteria are cultured. The results they achieved show that the MW-based sensor can present antibiotic susceptibility within 6 h, which is quicker than the current AST methods (15 h). In summary, compared with the popular AST methods, this new method requires less time and is more amenable to the slow-growing bacteria.

### Treating infection diseases

Currently, the popular methods of treating bacterial infections are photothermal therapy and photodynamic therapy, but these methods have poor penetration depth (Zhang et al., 2017; Wu et al., 2018b). Compared with solar light, MW can deeply penetrate tissues (Wang et al., 2017). A study (Ash et al., 2017) showed that in skin treatments, as the wavelength of light increases, so does the depth of light penetration, but only at a depth of millimeters. Mattsson et al. (2021) showed that microwaves can penetrate the muscle layer, which is a centimeter-level depth. With this ability, MW can be used to treat deep tissue infections. Recently, an increasing number of studies have focused on fabricating MW-sensitive nanomaterials to achieve MW specificity and selectivity against tumors (Wu et al., 2018b; Chen et al., 2020; Papini et al., 2020; Ricci et al., 2020; Weiss et al., 2020). However, the therapeutic potential of MW for bacterial infections should also receive more attention (Table 2).

**TABLE 2:** A summary of the destruction of bacteria by MW *in vivo*.

Experimental setup						
MW frequency (GHz)	Energy/power	Exposure temperature (°C)	Exposure time	Biological target/object	Effects	References
2.45	25 W	41–45	20 min/day for 7 days	Bone infection (Rat model)	MW can increase blood perfusion and kill bacteria.	Qi et al., 2019
2.45	0.1 W/cm^2^	50–55	20 min/day for 21 days	Osteomyelitis (Rabbit model)	This system, Fe3O4/CNT/Gent, is proven to efficiently target and eradicate MRSA-infected rabbit tibia osteomyelitis.	Qiao et al., 2020
-	8 W	55	5 min	Osteomyelitis (Rabbit model)	MW thermal effects and ROS resulted in death of the bacteria	Wei et al., 2021

Osteomyelitis as a challenging orthopedic disease has a protracted treatment (Rao et al., 2011). Some new treatment methods (hyperbaric oxygen therapy, pulsed electromagnetic fields, ultrasound, laser, and extracorporeal shockwave) are still unsatisfactory due to the impaired local blood supply and tissue perfusion (Emara et al., 2011; Inanmaz et al., 2014). A recent study (Qi et al., 2019) showed that the MW energy can be absorbed by deep tissue and increase the temperature, which increases the local blood flow, and more drug can be delivered to the lesion site. The results showed that MW irradiation (25 W, 20 min per day for 7 days) and cefuroxime reduce bacterial counts compared with cefuroxime alone (*p* < 0.05), as MW can increase blood perfusion and kill bacteria. Moreover, another study (Qiao et al., 2020) demonstrated the ability of MW combined with nanoparticles to treat osteomyelitis. They showed that, similar to photosensitizers in photodynamic therapy, MW therapy also has a sensitizer, Fe_3_O_4_/CNT as an MW sensitizer, and gentamicin as an antibiotic to treat MRSA-infected osteomyelitis. Fe_3_O_4_ and MW (2.45 GHz, 0.1 W/cm^2^) endow the nanoparticles with magnetic targeting and precise MW caloric therapy, which benefit the achievement of dramatic antibacterial effectiveness in deep tissue. Wei et al. (2021) developed a Prussian blue (PB) MOF as an MV-responsive material for the rapid treatment of osteomyelitis. PB MOFs are excellent at converting MV energy into heat and releasing iron ions. This results in increased permeability of the bacterial membrane to iron ions, which induces bacterial death through the production of highly harmful OH via the Fenton reaction. This of course results in a beneficial effect on superficial infections. In a mouse subcutaneous MRSA infection model, the novel nanoparticle PFG-IL/ZrO^2^-Ag@SiO_2_ combined with MW irradiation showed favorable effects both *in vitro* and *in vivo* (Wu et al., 2018b).

## Mechanisms

The mechanism of action of MW irradiation on bacteria is controversial (Shamis et al., 2012), and most scholars believe that there are two major mechanisms, thermal effects and non-thermal effects. One study (Cao et al., 2018) that compared the thermal effects of MW on cell membranes, cell walls, soluble chemical oxygen demand, enzyme inactivation and dysfunction, found that the non-thermal effects of MW (below 40°C) were more effective in destroying microorganisms. However, which type of cellular damage caused by each of these two mechanisms remains controversial. Therefore, we explain the mechanism by which MWs act on bacteria according to different types of cell damage.

### Membrane level

In terms of cell membrane damage, both thermal and non-thermal effects of MW exist. Meanwhile, this membrane damage is MW energy-dependent and MW working time-dependent (Table 3).

**TABLE 3:** A summary of the bacterial membrane damage by MW.

Experimental setup						
MW frequency (GHz)	Energy/power	Exposure temperature (°C)	Exposure time (min)	Biological target/object	Effects	References
18	1500 kW/m^3^	20–40	1	*E. coli*	Fluorescein isothiocyanate (FITC)- conjugated dextran (150 kDa) was taken up by the MW-treated cells, suggesting that pores had formed within the cell membrane.	Shamis et al., 2011
18	5.0 kW/kg	<40	1	Four cocci: *Planococcus maritimus, Staphylococcus aureus, S. aureus* and *S. epidermidis*	Exposing the bacteria to an EMF induced permeability in the bacterial membranes of all strains studied.	Nguyen et al., 2015
37.01	0.4 mW/cm2 20 mW	<40	–	*E. coli*	MW irradiation can transform the dynamic structural state of adsorbed water phases on biopolymer surfaces, which affect transport of ions K^+^ and H^+^ through the cellular membrane.	Kuznetsov et al., 2017
2.45	1800 W	85	5	*Bacillus Cereus*	MW results in the inactivation of *Bacillus cereus* by disrupting the cell membrane.	Cao et al., 2018
–	2000 W	100	2	*B. subtilis*	MW irradiation includes damage to the microbial cell wall.	Kim et al., 2008

Under low-energy and short-term MW irradiation, MW at sublethal temperature (40°C) can affect the permeability of bacterial cell membranes. A study (Shamis et al., 2011) on the effects of MW radiation showed that *E. coli* cells exhibited significantly different cell morphologies from negative controls after MW exposure, and CLSM showed that fluorescein isothiocyanate-conjugated dextran (150 kDa) was taken up by MW-treated cells. Another study (Nguyen et al., 2015) demonstrated this permeability of the bacterial membrane by detecting traces of leaking cytosolic fluid. Kuznetsov et al. (2017) conducted a study on *E. coli* and showed that MW irradiation (<10 mW/cm^2^) could transform the dynamic structural state of adsorbed water phases on biopolymer surfaces, which affect transport of K^+^ and H^+^ ions through the cellular membrane. However, this effect of the bacterial membrane appears to be temporary, as cell morphology can be restored, and bacterial survival rates reach as high as 84–88% (Shamis et al., 2011; Nguyen et al., 2015). This change in membrane permeability caused by MW is different from electroporation caused by PEF or high-energy electromagnetic fields (Garner et al., 2013). Electroporation requires a strong transmembrane potential, and the electroporation threshold decreases with the increase of the transmembrane thermal gradient (Gabrić et al., 2018; Garner, 2019). When the field strength is large enough, electroporation will be irreversible (Gabrić et al., 2018). One study (Katsuki et al., 2007) had also demonstrated that low-Hz PEF can cause changes in membrane permeability rather than electroporation. Recent studies have shown that MW induces a thermal gradient across the membrane (Garner et al., 2013), which reduces the transmembrane potential for microwave-induced electroporation (Song et al., 2017; Garner, 2019), which may explain NAME when nanotubes are combined with MW (Gao et al., 2019). It is possible that the nanotubes exacerbate the thermal gradient across the membrane under the action of MW.

Under high-energy and long-term MW irradiation, MWs can disrupt the integrity of the bacterial cell membrane and inactive bacteria. Cao et al. (2018) used Scanning Electron Microscope (SEM) analysis to show that MW irradiation (1800 W, 85°C for 5 min), led to severe morphological disruption of the cell membrane, resulting in the release of nuclear components and proteins from the cytoplasm. In Kim’s study (Kim et al., 2008), the authors attributed this release of nuclear components and proteins to disruption of the cytoplasmic membrane by MW (2000 W, for 2 min), which is not observed following conventional heating (boiling, for 10 min).

In summary, we can find that when MW acts on bacteria with lower power and shorter acting time at non-lethal temperature, the membrane permeability of bacteria is changed. This provides new ideas for the development of new methods for MW-assisted gene therapy, drug delivery, and substance extraction. When MW acts on bacteria with higher power and longer action time at lethal temperature, the bacterial membrane and cytoplasmic membrane are irreversibly damaged, which can directly lead to bacterial death. This indicates that it is feasible to use MW to kill bacteria and disinfect in the fields of biology and hygiene, and it has been reported (Hoff et al., 2021; Tilley et al., 2021) that MW systems have been implemented for the killing of pathogens in the air.

### Metabolic level

One study (Cao et al., 2018) showed that the expression of 23 *Bacillus cereus* proteins was regulated after MW (2000 W, for 2 min) treatment. These proteins include carbohydrate metabolism-related enzymes, such as L-lactate dehydrogenase, transaldolase and malate dehydrogenase, fructose bisphosphate aldolase and triose phosphate isomerase. In addition, all enzymes were involved in amino acid or protein metabolism, except histidine dipeptidase. Moreover, Mn-SOD, an enzyme that can effectively scavenge free radicals generated by metabolism and protect cells against oxidative damage, was downregulated. These effects were attributed to megawatt radiation.

Reactive oxygen species (ROS) play a very important role in bactericidal activity, which occurs through a decline in antioxidant mechanisms, such as a drop in glutathione (GSH) levels. Shaw et al. (2021) showed that MW exposure induced the production of intracellular ROS and decreased GSH levels in *E. coli* and *S. aureus*, which contributed to the inactivation of both bacteria.

### Genetic level

Some studies have suggested that MW functions at the genetic level (Ruediger, 2009; Torgomyan and Trchounian, 2013). Research has shown that (Tong et al., 2016), after combined MW pretreatment, the ARG concentration was lower than that after sludge anaerobic digestion alone. Another study (Shaw et al., 2021) conducted further experiments of genetic damage of MW on bacteria, and showed that MW irradiation caused oxidative stress-mediated DNA damage. Furthermore, when *E. coli* was exposed to MW, the transcriptional gene expression levels of six oxidative stress genes were reduced, including SoxS, OxyR, KatG, RpoE, GroES, and DnaK.

## Prospects and challenges

### Perspectives

*In vitro*, compared with traditional autoclaving, MW irradiation can generate highly concentrated energy pulses in a limited time, directly penetrating and heating the material without any intermediate heat transfer medium (Vogl et al., 2011; Wang et al., 2012). Compared with chemical sterilization, MW sterilization is more environmentally friendly (Goel et al., 2014). Given that high-power, long-term MW can effectively destroy bacterial cell membranes to kill bacteria (Kim et al., 2008; Nguyen et al., 2015; Park et al., 2017; Martinez-Serna et al., 2021), MW has shown good development potential in the sterilization and biofilm destruction of laboratory and medical equipment (Yezdani et al., 2015), foods (Song and Kang, 2016; Cho and Chung, 2020), and environment (Mawioo et al., 2016; Tong et al., 2016). Recently, many microwave systems (Hoff et al., 2021; Tilley et al., 2021) have been used for the inactivation of airborne pathogens, and the effect of killing pathogens is remarkable, which gives us hope for the development and application of microwave *in vitro* sterilization. Since MW is extremely sensitive to changes in surrounding conductivity caused by bacterial growth (Mohammadi et al., 2020; Jain et al., 2021a,b). And low-power, short-term MW can alter bacterial cell membrane permeability, with the help of nanotubes, MW radiation can induce NAME on bacterial cell membranes (Gao et al., 2019). These capabilities lead to a faster method for bacterial monitoring, identification and antibiotic susceptibility than traditional methods. This can help optimize antibiotic prescribing, reduce resistance and prevent the spread of multidrug-resistant pathogens. In addition, the change of bacterial cell membrane permeability makes it possible to extract bacterial intracellular substances and intracellular delivery of drugs. In recent years, microwave imaging technology has provided a high-contrast, non-invasive, and rapid imaging method (Modiri et al., 2017; Lin, 2021). Tissue water content is a key factor in microwave imaging, and tissue edema and high blood flow at the site of infection make it possible to diagnose and monitor infections using microwave imaging.

*In vivo*, MW is a promising treatment modality with the advantages of simple operation, less invasiveness, deep penetration depth, local controllability, high heating efficiency, and a wide heating area (Gu et al., 2011; Vogl et al., 2011). At present, MW has been widely used in clinical treatment for tumors, warts, underarm odor, hypertrophy of turbinates and various non-infectious inflammations (Hsu et al., 2017; Fu et al., 2019; Jackson et al., 2020; Llovet et al., 2021). This proves that MW is safe under reasonable operation and has certain clinical application value. However, the application of MW to human treatment of infectious diseases still needs further exploration and research.

For local body surface infection, local MW radiation can be given directly, combined with antibiotics, which can not only promote local blood circulation, but also the drug can better enter the bacteria with altered membrane permeability, which is beneficial to the killing of bacteria. For local deep infection (such as: joint cavity infection, periprosthetic infection, etc.), if you want to kill bacteria through simple MW, it will cause damage to normal human tissues. At present, there have been relevant animal experimental studies trying to combine MW with other treatment methods to carry out effective antibacterial *in vivo* (Qi et al., 2019; Qiao et al., 2020). In recently years, photodynamic and photothermal therapy related research on *in vivo* sterilization is relatively popular. With the development of MW sensitizers, MW is expected to become an excitation switch for microwave hyperthermia and microwave dynamic therapy. When the drug reaches the local area (targeted at the infection site or enrichment caused by local temperature increase), MW radiation is applied to achieve thermal effect sterilization and biodynamic sterilization. Cause MW sensitizers can easily convert microwave energy into heat, the application of these materials can reduce side effects on surrounding objects and improve microwave heating efficiency (Gu et al., 2011; Wang et al., 2012; Wu et al., 2018a), and the microwave sensitizer promotes MW to produce harmful active substances or in a similar way to electroporation, thereby sterilizing. Due to these advantages, MW can be used to treat deep-seated infections (Xiang and Chen, 2019). However, the biocompatibility of MW sensitizers should be further investigated before *in vivo* application.

In summary, low-energy, short-duration microwave irradiation can lead to the permeabilization of bacterial membranes (Nguyen et al., 2015). This capability could be used to develop a new bacterial membrane permeation technology by which the sensitivity of bacteria to antibiotics can be increased, and bacterial drug delivery, bacterial gene therapy, and biomedical engineering can be enabled. In the treatment of human infectious diseases, MW can increase blood flow through the thermal effect, promote the local accumulation of drugs and enter the bacterial cell membrane by changing the bacterial membrane permeability. And the sterilization ability of MW hyperthermia and MW dynamic therapy can be enhanced with the assistance of MW sensitizers.

### Challenges

There are some hurdles that need to be addressed in the application of MW. First, electromagnetic interference is a serious problem that can cause electrical equipment to fail, affecting lives (Jang et al., 2020), and is also recognized as the fourth largest public nuisance after air pollution, water pollution and noise pollution (Saini et al., 2009; Green and Chen, 2019). Fortunately, an increasing number of researchers have devoted themselves to the field of MW absorption and electromagnetic interference shielding and have achieved many excellent research results (Quan et al., 2017; Wang L. et al., 2021). Second, the temperature control of MW irradiation is limited, which poses a risk for *in vivo* therapy because MW interacts with all polar molecules (Martinez-Serna et al., 2021; Shaw et al., 2021). At present, there is still a lack of relevant research on the effects of MWs of different frequencies on the human body, and further research is needed. However, it is undeniable that MW therapy has achieved great success in tumors *in vivo* (Chen et al., 2020; Cazzato et al., 2021). Finally, knowledge of the mechanisms and the non-thermal effects of MW is limited. Therefore, for the further application of MW, it is necessary and urgent to study the MW-related mechanisms in depth. Despite these limitations, with the widespread use of MW (Nagahata and Takeuchi, 2019; Yang and Park, 2019; Mirzadeh et al., 2020), these challenges will eventually be resolved, and the widespread application of MW in the field of bacteria will be realized.

## Author contributions

ZZ: conceptualization and writing – original draft. JW: writing – original draft. YH: writing – review and editing. LW: conceptualization and writing – review and editing. All authors contributed to the article and approved the submitted version.
